# Impact of helical organization on the photovoltaic properties of oligothiophene supramolecular polymers†
†Electronic supplementary information (ESI) available: Synthesis and characterization of **3** and **4**, UV-vis spectra, solar cell device properties and AFM images. See DOI: 10.1039/c7sc05093c


**DOI:** 10.1039/c7sc05093c

**Published:** 2018-03-12

**Authors:** Hayato Ouchi, Takahiro Kizaki, Masaki Yamato, Xu Lin, Nagahiro Hoshi, Fabien Silly, Takashi Kajitani, Takanori Fukushima, Ken-ichi Nakayama, Shiki Yagai

**Affiliations:** a Division of Advanced Science and Engineering , Graduate School of Science and Engineering , Chiba University , 1-33 Yayoi-cho, Inage-ku , Chiba 263-8522 , Japan; b Department of Organic Device Engineering , Graduate School of Science and Engineering , Yamagata University , 4-3-16 Jonan , Yonezawa , Yamagata 992-8510 , Japan; c Department of Organic Materials Science , Graduate School of Organic Materials Science , Yamagata University , 4-3-16 Jonan , Yonezawa , Yamagata 992-8510 , Japan; d Department of Material and Life Science , Graduate School of Engineering , Osaka University , 2-1 Yamadaoka , Suita , Osaka 565-0871 , Japan; e Department of Applied Chemistry and Biotechnology , Graduate School of Engineering , Chiba University , 1-33 Yayoi-cho, Inage-ku , Chiba 263-8522 , Japan; f TITANS , SPEC , CEA , CNRS , Université Paris-Saclay , CEA Saclay , F-91191 Gif sur Yvette , France; g Laboratory for Chemistry and Life Science , Institute of Innovative Research , Tokyo Institute of Technology , 4259 Nagatsuta, Midori-ku , Yokohama 226-8503 , Japan; h RIKEN SPring-8 Center , 1-1-1 Kouto , Sayo , Hyogo 679-5148 , Japan; i Institute for Global Prominent Research (IGPR) , Chiba University , 1-33 Yayoi-cho, Inage-ku , Chiba 263-8522 , Japan . Email: yagai@faculty.chiba-u.jp ; Fax: +81-(0)43-290-3401 ; Tel: +81-(0)43-290-3169

## Abstract

Helical self-assembly of functional π-conjugated molecules offers unique photochemical and electronic properties in the spectroscopic level, but there are only a few examples that demonstrate their positive impact on the optoelectronic device level. Here, we demonstrate that hydrogen-bonded tapelike supramolecular polymers of a barbiturated oligo(alkylthiophene) show notable improvement in their photovoltaic properties upon organizing into helical nanofibers. A tapelike hydrogen-bonded supramolecular array of barbiturated oligo(butylthiophene) molecules was directly visualized by STM at a liquid–solid interface. TEM, AFM and XRD revealed that the tapelike supramolecular polymers further organize into helical nanofibers in solution and bulk states. Bulk heterojunction solar cells of the helical nanofibers and soluble fullerene showed a power conversion efficiency of 4.5%, which is markedly high compared to that of the regioisomer of butyl chains organizing into 3D lamellar agglomerates.

## Abstract 2

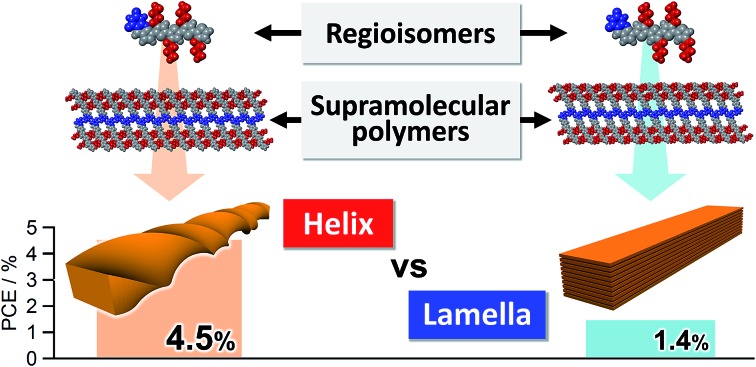
Higher order structures of semiconducting supramolecular polymers have a huge impact on their BHJ-OPV device performance.

## Introduction

Helical secondary structure plays a central role in the organization processes of polypeptides, and one of the topological advantages of this motif might be its discrete nature that suppresses unfavorable aggregation of polypeptide chains. Inspired by this biological example, supramolecular chemists have devoted much effort to create helical nanoarchitectures composed of functional π-conjugated molecules with the expectation that such helical structures should show unique optoelectronic properties.^1^ However, there are only a few examples that demonstrate the positive impact of helical structures on the performance of optoelectronic devices.^2^

Bulk heterojunction (BHJ) organic photovoltaic (OPV) devices using small molecules have attracted increasing attention due to well-defined molecular structures that can facilitate exploration of structure–property relationships and guide design rules towards better OPV devices.^3^ In the optimization process of BHJ-OPV device fabrication, control over nanostructures of semiconducting donor and acceptor molecules and their phase separation in the active layer is of primary importance because they are directly related to charge separation and transportation efficiencies.^4^ Hydrogen bond is one of the powerful tools to precisely control molecular self-organization in the nanoscopic level.^5^ Several research groups have already applied hydrogen-bonding small-molecule semiconductors for BHJ-OPV devices, but power conversion efficiencies (PCEs) of the devices were generally low due to the requirement of installing solubilizing yet non-conducting long alkyl chains into molecular scaffolds.^6^

As exceptional examples of hydrogen-bonding small-molecule semiconductors for BHJ-OPV devices, we have shown the hierarchical self-assembly and photovoltaic properties of barbiturated oligo(hexylthiophene)s.^7^ For example, we have found by scanning tunneling microscopy (STM) that 3-hexylthiophene derivative **1** selectively forms hydrogen-bonded supermacrocyclic hexamers (rosettes) at the liquid–solid interface.^8^ The rosettes further organize upon solution casting into nanorods that can be visualized by atomic force microscopy (AFM). Reflecting their unique rodlike nanostructures free from exterior non-conducting alkyl chains, BHJ-OPV devices of **1** and a solution-processable fullerene derivative (PC_71_BM, [6,6]-phenyl-C_71_-butyric acid methyl ester) achieved a PCE of 3.01%, which was exceptionally high among the devices using hydrogen-bonding semiconductors.^8^,^9^ In sharp contrast, a regioisomeric 4-hexylthiophene analogue **2** preferentially formed infinite tapelike hydrogen-bonded supramolecular polymers at the liquid–solid interface.^10^ Because the tapelike supramolecular polymers densely organize into a lamellar structure, BHJ-OPV devices of **2** did not show PCE above 1.5% due to macroscopic phase separation with soluble fullerene derivatives.

**Fig. 1:**
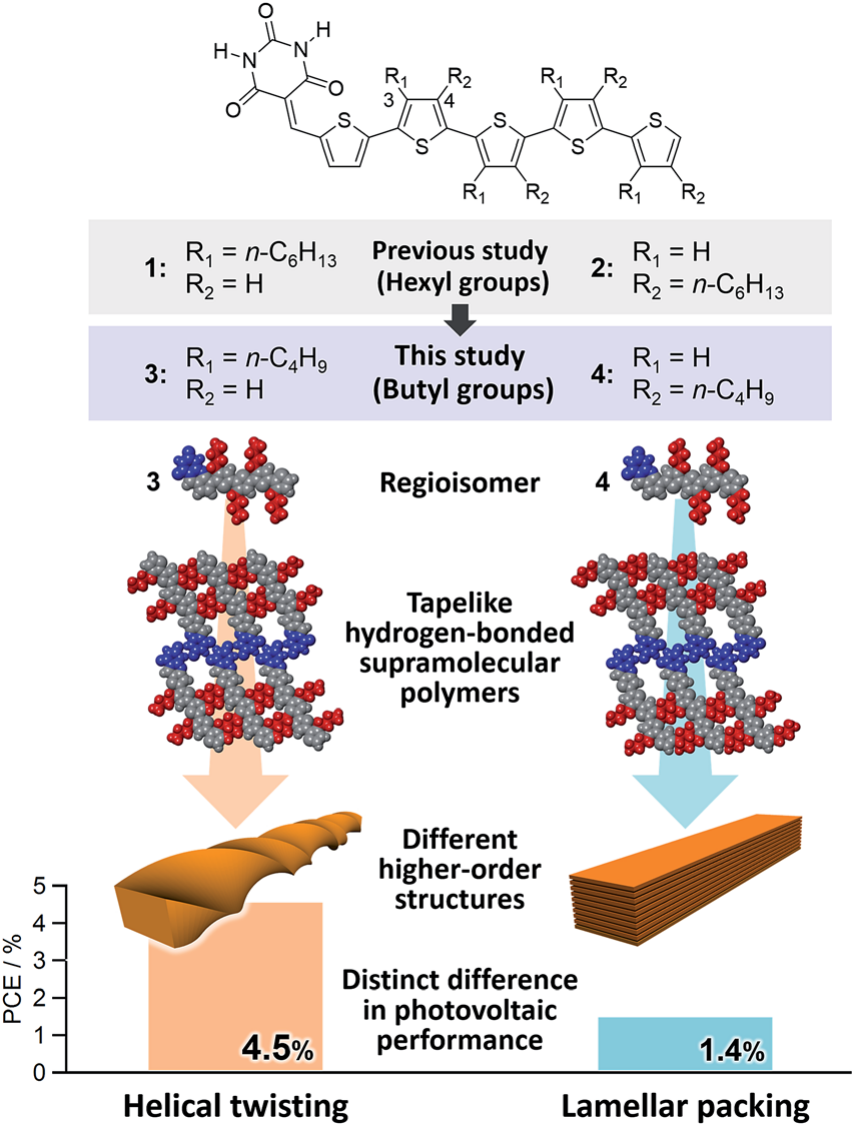
Molecular structures of barbiturated oligothiophenes **1**, **2**, **3**, and **4**, and their hydrogen-bonded motifs and self-assembled nanostructures of **3** and **4** with the best PCEs of the optimized BHJ solar cells.

Based on the above results, we have undertaken further improvement of device performance of our hydrogen-bonded oligothiophene semiconductors by shortening the alkyl chains from hexyl to butyl (**3** and **4**, Fig. 1). To our surprise, BHJ-OPV devices of **3** and **4** displayed remarkably different performance (ΔPCE > 3.0%) although both **3** and **4** formed tapelike hydrogen-bonded supramolecular polymers at the liquid–solid interface. We thus investigated the hierarchical organization processes of **3** and **4**, and revealed that the distinct performance is attributed to the difference in the higher order self-assembly pathway of tapelike supramolecular polymers either through helical twisting or continuous lamellar stacking.

## Results and discussion

### Hydrogen-bonding motifs

**Fig. 2:**
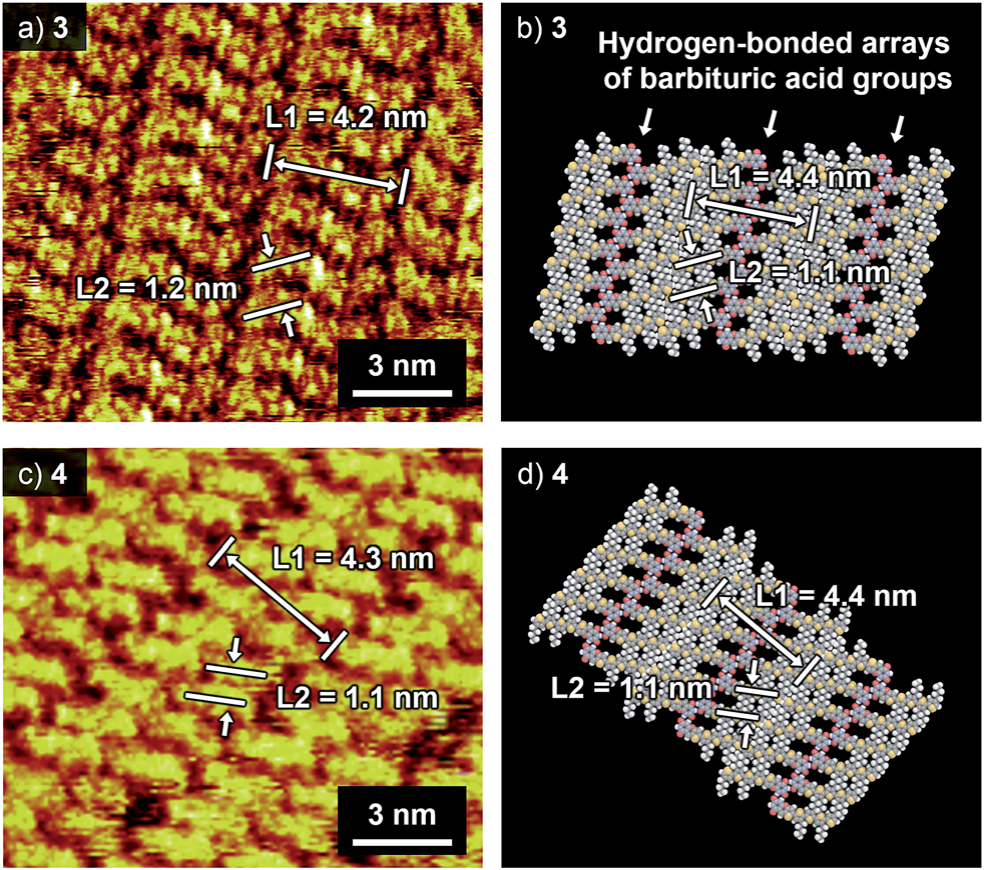
(a and c) STM images of (a) **3** and (c) **4** at the 1-phenyloctane–HOPG interface. Tunneling current (*I*_t_) = 9 pA, bias voltage (*V*_s_) = +0.65 V. The concentration of the solution is 5 × 10^–6^ M. (b and d) Molecular models of two-dimensionally arranged tapelike supramolecular polymers of (b) **3** and (d) **4** based on the STM images.

Compounds **3** and **4** were synthesized according to Scheme S1† and characterized by ^1^H NMR spectroscopy, mass spectrometry, and elemental analysis (see the ESI†). To reveal the hydrogen-bonding motif preferred by the two compounds, their self-assembly was investigated at the liquid–solid interface by means of STM. Fig. 2a and c show typical STM images of **3** and **4** at the interface between 1-phenyloctane and highly oriented pyrolytic graphite (HOPG).^11^ For both compounds, lamellarly organized molecular arrangements have been visualized.^12^ Molecular modelling showed that the interlayer spacing L1 and the intermolecular distance L2 in the STM images coincide well with the length of the hydrogen-bonded dimeric unit and the distance between the neighbouring dimeric units that are also held together by hydrogen bonds (Fig. 2b and d). The formation of a tapelike motif by **3** despite the fact that the hexyl derivative **1** prefers a rosette motif is presumably due to the lack of interactions between alkyl chains that stabilize rosette structures.^10^

### Nanostructures

**Fig. 3:**
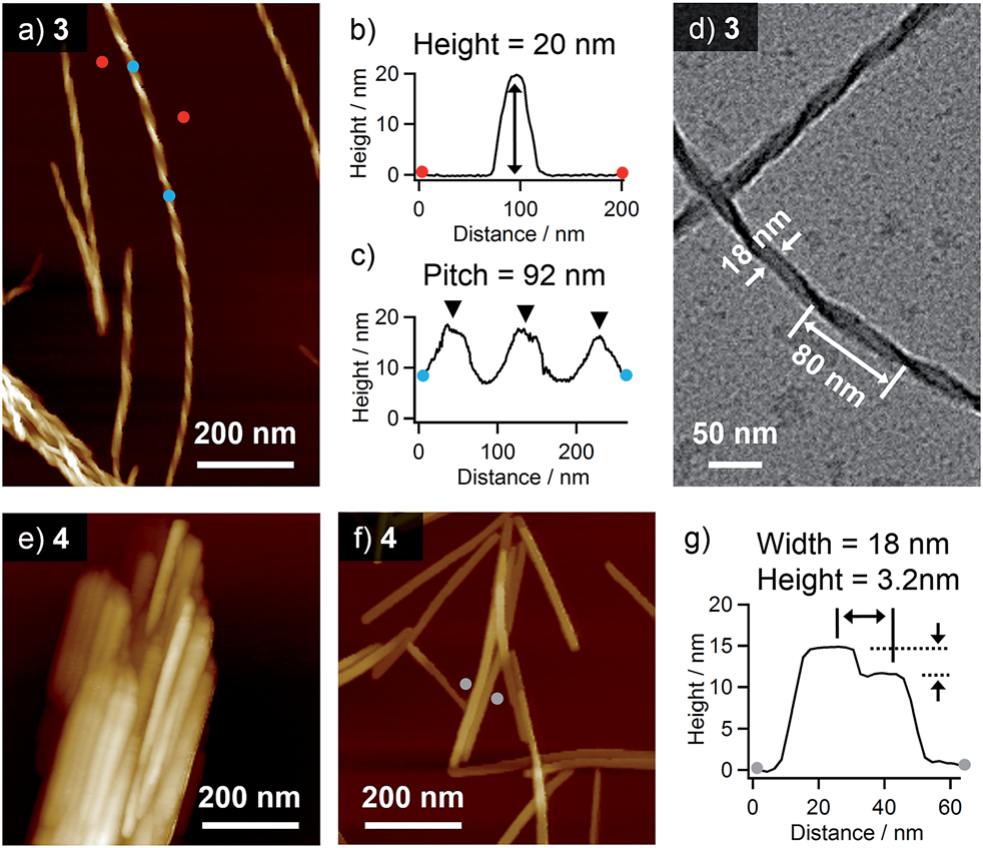
(a) AFM and (d) TEM images of helical nanofibers of **3** formed in toluene (*c* = 1 × 10^–3^ M). (b and c) Cross-sectional analysis between (b) red dots (for height) and (c) blue dots (for pitch) in the image (a). (e) AFM image of the lamellae of **4** formed in toluene (*c* = 5 × 10^–4^ M). (f) AFM image of thin film prepared by drop-casting toluene solution of **4** (*c* = 1 × 10^–4^ M) onto HOPG. (g) Cross-sectional analysis between gray dots (for width and height) in the image (f).

Higher-order organizations of **3** and **4** were investigated in a less polar solvent, toluene. Upon cooling hot solutions (**3**: *c* = 1 × 10^–3^ M, **4**: *c* = 5 × 10^–4^ M, *ca.* 100 °C) of **3** and **4** to room temperature, both compounds afforded precipitates. AFM observation of the precipitate of **3** revealed the formation of uniform helical nanofibers with a diameter of 20 nm and a pitch of 92 nm (Fig. 3a–c). By transmission electron microscopy (TEM), almost similar helical morphology with a comparable diameter and a pitch (80 nm) was confirmed (Fig. 3d).

In contrast, the AFM image of the precipitate of **4** displayed heavily bundled rodlike structures (Fig. 3e). To investigate the detailed morphology of the elementary structure, a more diluted solution of **4** (*c* = 1 × 10^–4^ M) was drop-cast onto HOPG, and the solvent was slowly evaporated to organize molecules. By this way, we could see dispersed rodlike structures with a width of *ca.* 18 nm and inhomogeneous heights of 3.2–11 nm (Fig. 3f, g and S1†). The flat surface of rodlike structures indicates the absence of a helical higher-order structure, and their inhomogeneous thickness rather suggests the formation of a layered structure by the stacking of tapelike supramolecular polymers.

### Powder XRD

**Fig. 4:**
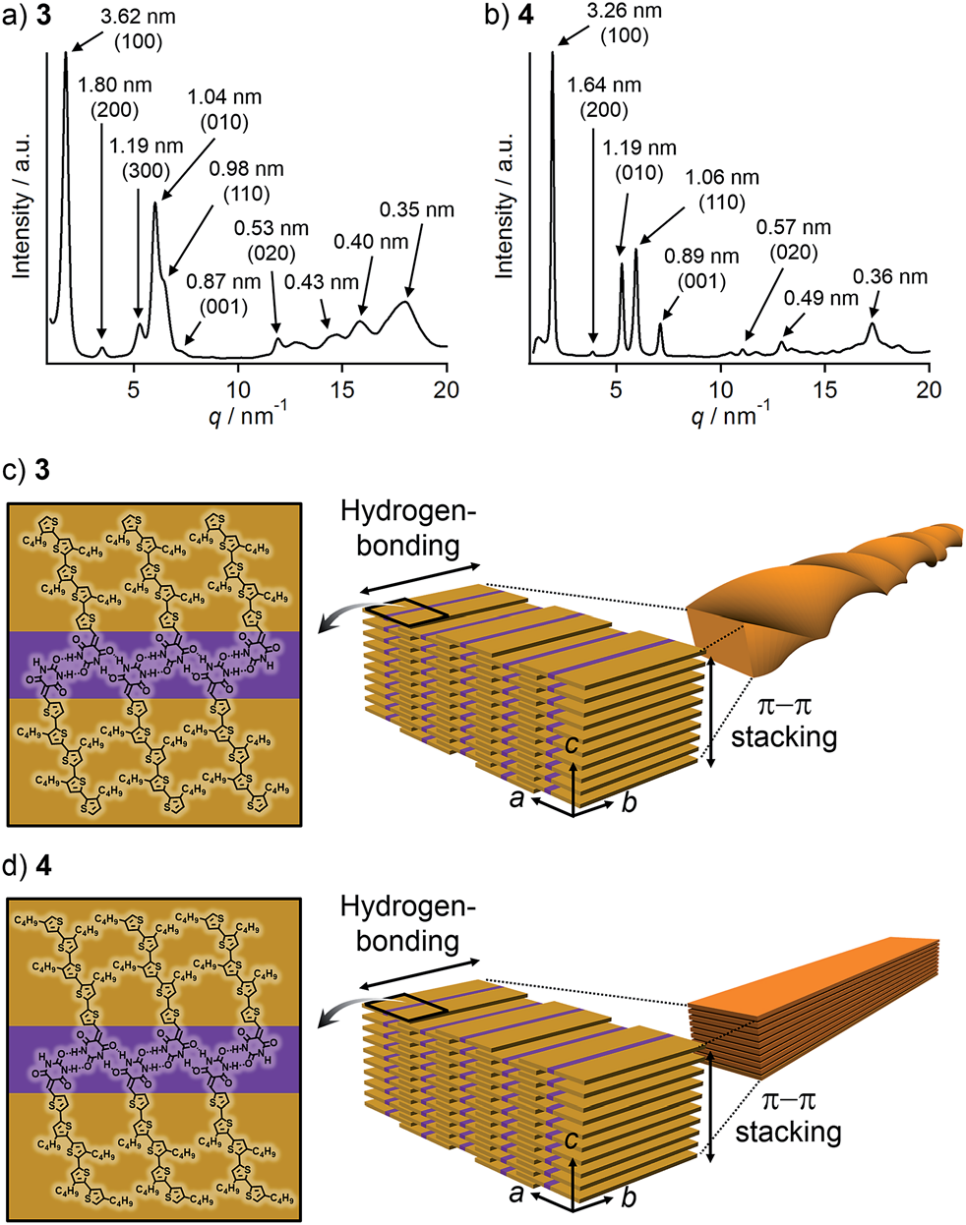
(a and b) PXRD patterns of precipitates formed from the toluene solution of (a) **3** and (b) **4** in a glass capillary (diameter: 1.0 mm). Values in parenthesis denote Miller indices. (c and d) Schematic representation of the proposed packing structures of (c) **3** in helical nanofibers and (d) **4** in lamella.

To corroborate that the above one-dimensional nanostructures are formed by the hierarchical organization of the tapelike supramolecular polymers observed by STM, powder X-ray diffraction (PXRD) measurements were performed for the precipitates. The PXRD pattern of the precipitate of **3** displayed seven peaks in the small-angle region (Fig. 4a). These peaks can be considered as two sets of diffractions with the reciprocal of 1 : 2 : 3 ratio (*d* = 3.62/1.80/1.19 and *d* = 1.04/0.53 nm). Accordingly, the presence of two different structural elements of 3.62 nm and 1.04 nm periodicities can be proposed, which is characteristic of a 2D rectangular lattice. These five peaks can be thus assigned to the diffractions from the (100), (200), (300), (010), and (020) planes of a 2D rectangular lattice (space group: *P*2*m*, lattice parameters: *a* = 3.6 nm, *b* = 1.0 nm) in the order of decreasing *d*-spacing. While the remaining peak at *d* = 0.98 nm can be assigned to the diffraction from the (110) plane, another one at *d* = 0.87 nm could be attributed to a structural periodicity element along the *c*-axis (Fig. 4c). Likewise, the PXRD pattern of the precipitate of **4** could be characterized as a 2D rectangular lattice (space group: *P*2*m*, lattice parameters: *a* = 3.3 nm, *b* = 1.2 nm, Fig. 4b). Overall peaks were sharper than that of **3**, suggesting a higher crystallinity of **4**. This is also supported by the melting point of **4** (221 °C), which is higher than that of **3** (184 °C) as revealed by differential scanning calorimetry (DSC) (Fig. S2†).

Taking the PXRD as well as the aforementioned STM results into consideration, we proposed the packing structures of **3** and **4** as shown in Fig. 4c and d, respectively. Tapelike hydrogen-bonded supramolecular polymers stack mainly *via* π–π stacking between oligothiophene moieties, and the stacked tapes laterally organize by interdigitating exterior butylthiophene moieties. The lattice parameter *b* by XRD feasibly corresponds to the intermolecular distance L2 between dimeric units in the STM images, while the lattice parameter *a* could be correlated with the width L1 of the supramolecular chain (Fig. 2a) by assuming that the chains are stacked in a slipped brick-like motif.^10^ In the case of **3**, the resulting finite stacks of supramolecular polymers twist like amyloid β-fibrils to form helical nanofibers.^13^,^14^ In this model, helical nanofibers can grow in their lengths through the elongation of hydrogen-bonded chains and in their thicknesses through the stacking of oligothiophene moieties, respectively. On the other hand, supramolecular polymers of **4** organize into crystalline multilamellar structures (Fig. 4d). Why do **3** and **4** organize into the distinct nanostructures *via* the formation of the same hydrogen-bonded tapelike supramolecular polymers? This can be explained by the steric hindrance between barbituric acid and the nearest butyl chain. Because butyl chains of **3** in the supramolecular polymer are directed to the central barbituric acid array, steric hindrance between barbituric acid and the nearest butyl chain of **3** causes the twisting of the oligothiophene backbones. The conformational distortion of individual building blocks may lead to a higher-order twisting of the tapelike supramolecular polymer.^10^

### BHJ-OPV devices

**Fig. 5:**
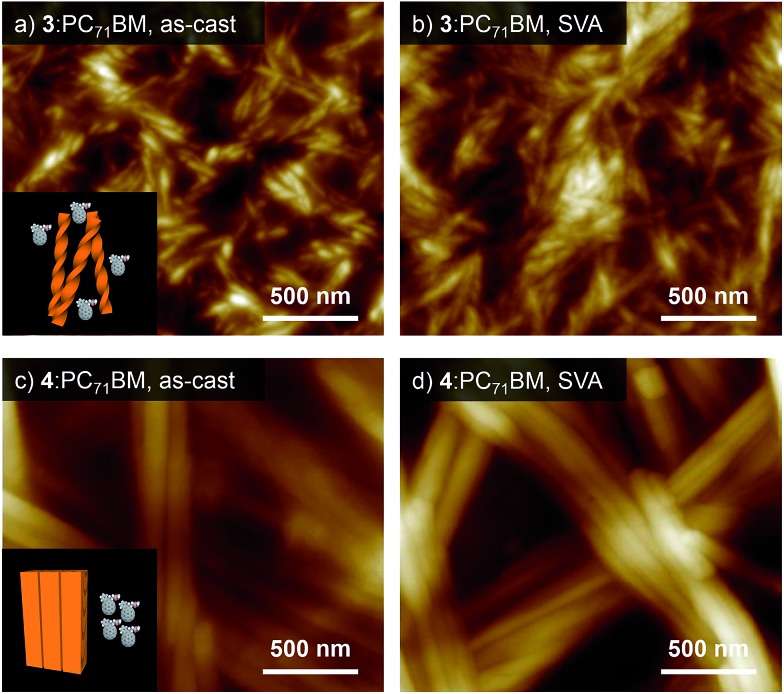
(a–d) AFM images of (a and b) **3**:PC_71_BM and (c and d) **4**:PC_71_BM films (a and c) before and (b and d) after SVA using CS_2_ for 80 s. Inset of (a) and (c): schematic illustration of the morphologies of **3**:PC_71_BM and **4**:PC_71_BM.

The significantly different organized structures of **3** and **4** despite minor difference in their molecular structures motivated us to evaluate their photovoltaic properties in BHJ-OPV devices. BHJ films were prepared by spin-coating the chloroform solution of a 1 : 1 mixture of our hydrogen-bonded oligothiophene and PC_71_BM. The resulting films (denoted as **3**:PC_71_BM and **4**:PC_71_BM, respectively) showed absorption bands in the range of 300–700 nm (Fig. S4†). PXRD analysis confirmed that both **3** and **4** can organize into similar lamellar structures in the presence of PC_71_BM (Fig. S5†). While the diffraction pattern assigned to a 2D rectangular lattice of **4** was not affected by PC_71_BM, **3** showed a decrease of the lattice parameter *a* (3.62 → 3.30 nm) by PC_71_BM. The distinct influence of PC_71_BM on the molecular packing implies that phase-separation between the oligothiophenes and PC_71_BM occurs at different levels in the two systems, namely, helically organized supramolecular polymers of **3** should be more miscible with PC_71_BM due to the discrete nature of the helical secondary structure, allowing certain degree of structural organization in the PC_71_BM matrix. The different levels of phase-separation were unequivocally shown by morphology observation of the film surface by AFM. The AFM image of **3**:PC_71_BM shows fibrous structures with widths of around 30 nm (Fig. 5a),^15^ whereas that of **4**:PC_71_BM featured fibers with submicrometer width (Fig. 5c). Reflecting these morphological differences, the BHJ-OPV devices fabricated using the as-cast film of **3**:PC_71_BM and **4**:PC_71_BM showed a clear difference in PCE (2.10 and 1.19%, respectively, Table 1).^16^ Obviously, charge separation would occur more efficiently in **3**:PC_71_BM wherein the phase-separation occurs in the scale less than 100 nm. This notion was also supported by a significant difference in their external quantum efficiency (EQE) in the range of 400–600 nm (**3**:PC_71_BM: 52.6%, **4**:PC_71_BM: 24.3%, Fig. S7†).

**Table 1:** Effect of solvent vapor annealing (SVA) with CS_2_ on the photovoltaic properties of **3**:PC_71_BM and **4**:PC_71_BM BHJ solar cells

BHJ films	SVA time [s]	*J*_sc_ [mA cm^–2^]	*V*_oc_ [V]	FF [%]	PCE [%]
**3**:PC_71_BM	As-cast	6.63 ± 0.11	0.86 ± 0.02	36.9 ± 0.7	2.10 ± 0.09
	40	9.57 ± 0.11	0.76 ± 0.01	60.7 ± 0.9	4.38 ± 0.07
	80	9.73 ± 0.20	0.74 ± 0.00	62.2 ± 0.8	4.50 ± 0.09
	120	9.31 ± 0.21	0.75 ± 0.01	60.5 ± 1.3	4.19 ± 0.10
**4**:PC_71_BM	As-cast	3.53 ± 0.23	0.75 ± 0.01	45.2 ± 0.2	1.19 ± 0.10
	40	3.98 ± 0.14	0.69 ± 0.02	46.9 ± 0.7	1.27 ± 0.03
	80	4.45 ± 0.10	0.34 ± 0.04	33.5 ± 1.3	0.51 ± 0.08
	120	3.95 ± 0.13	0.70 ± 0.02	50.2 ± 0.4	1.39 ± 0.01

In our previous study on **1**:PC_71_BM, it was shown that thermal annealing of the as-cast BHJ film improves the device performance (PCE = 1.29 → 3.01%) by growing nanorods formed by rosettes.^8^ For **3**:PC_71_BM, however, thermal annealing was found to be ineffective in improving the performance (2.10 → 1.82%, Table S1†), and this is not surprising because helical nanofibers have already “matured” through the solution casting (Fig. 5a). For **4**:PC_71_BM, thermal annealing at 80 °C only slightly increased the device performance (PCE = 1.19 → 1.33%, Table S1†), while further raising the annealing temperature to 110 °C seriously reduced the PCE from 1.19 to 0.26% with a large drop of short circuit current density (*J*_sc_: 3.53 → 0.81 mA cm^–2^) due to the overgrowth of the donor and acceptor phases.

As an alternative method to reorganize molecular packing, we applied solvent vapor annealing (SVA).^17^ To our surprise, SVA with CS_2_ improved the performance of solar cells only fabricated with **3**:PC_71_BM (Table 1) although noticeable surface morphology change was observed neither for **3**:PC_71_BM nor for **4**:PC_71_BM by SVA (Fig. 5b and d). The current–voltage (*J*–*V*) curves of the **3**:PC_71_BM devices fabricated without CS_2_ and with CS_2_ vapor treatment illustrated a remarkable increase in *J*_sc_ from 6.63 to 9.73 mA cm^–2^ with a moderate decrease in *V*_oc_ from 0.86 to 0.74 V by SVA (Fig. S8a†).^18^ Reflecting these changes in the device properties, the PCEs increased considerably from 2.10% to 4.50% with an increase of fill factor (FF) from 36.9 to 62.2%. Meanwhile, SVA with CS_2_ was not effective in improving the device performance of **4**:PC_71_BM (Table 1 and Fig. S8b†). It is conceivable that a thermodynamically stable crystalline packing is already achieved for **4**:PC_71_BM during solution processing due to the higher crystallinity of **4**.

**Fig. 6:**
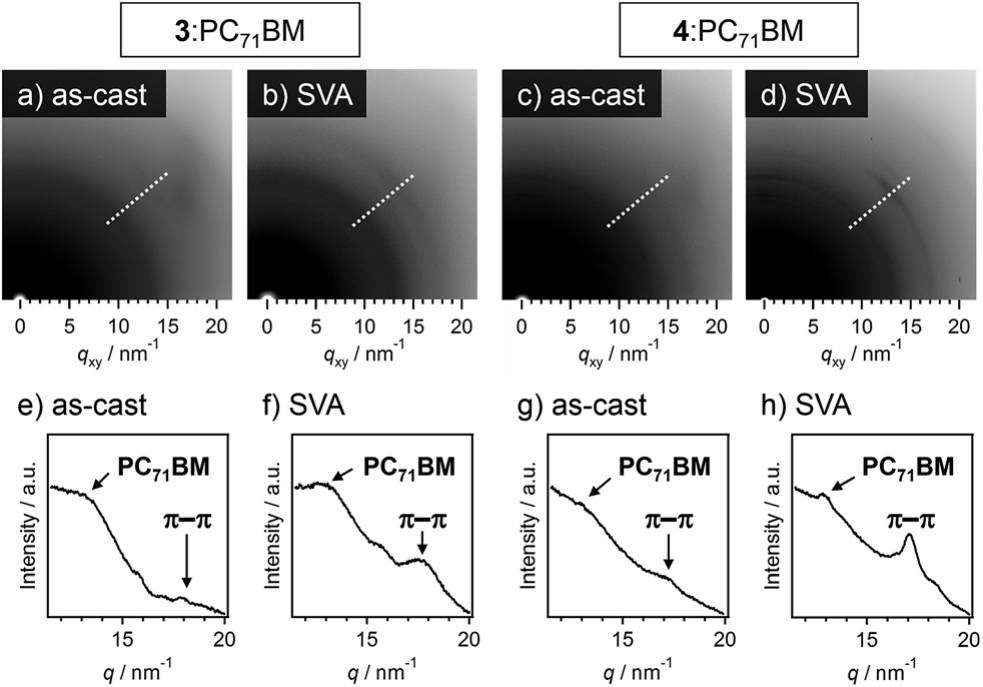
(a–d) GI-XRD images of (a and b) **3**:PC_71_BM and (c and d) **4**:PC_71_BM films. Images (a and c) were taken in the as-prepared state, and images (b and d) were taken after SVA using CS_2_ for 80 s. (e–h) Radial profiles along the direction shown by white dashed lines in images (a–d), respectively, to pick up diffractions associated with PC_71_BM and π–π stacked oligothiophene moieties.

In grazing incidence X-ray diffraction (GI-XRD) measurements of **3**:PC_71_BM and **4**:PC_71_BM, the diffraction ring corresponding to the π–π stacking of oligothiophene moieties (*d* = 0.35 nm) became clearer after SVA (Fig. 6). Hence, SVA seems to effectively increase the structural ordering of oligothiophene units for both the mixtures. To gain more insight into the effect of SVA, hole mobilities in **3**:PC_71_BM and **4**:PC_71_BM were evaluated by the space-charge limited current (SCLC) method (Table S2†); the change of hole mobilities in both systems is less than one-order of magnitude. In **3**:PC_71_BM, however, the hole mobility increased from 6.6 × 10^–6^ to 1.2 × 10^–5^ cm^2^ V^–1^ s^–1^ by SVA for 80 s. In **3**:PC_71_BM, a sufficient quantity of free charge carrier should be generated in the finely phase-separated nanostructures, the improvement of hole mobilities might be directly reflected in PCE. In the case of **4**:PC_71_BM, the hole mobility also showed a moderate increase by SVA for 80 s (1.2 × 10^–3^ → 4.3 × 10^–3^ cm^2^ V^–1^ s^–1^). Why this improvement in the hole mobility is not reflected in PCE is due to the macroscopic phase separation that is not capable of generating sufficient charge carriers upon light irradiation. Thus, the extended supramolecular organization of **4** is a bottle-neck to improve the device performance of **4**:PC_71_BM.^19^

## Conclusions

In conclusion, we have demonstrated that hydrogen-bonded tapelike supramolecular polymers of barbiturated oligothiophene **3** hierarchically organize into helical nanofibers. This organization pathway was maintained in the presence of a soluble fullerene derivative, affording reasonable BHJ nanostructures that can realize the PCE of 4.5% in BHJ solar cells. This performance is outstanding among those based on hydrogen-bonding small-molecular materials, and very contrastive to the result of oligothiophene **4** that also forms tapelike supramolecular polymers but further organizes into 3D lamellar agglomerates. Hence, the present study demonstrates that not only controlling primary molecular arrays but also regulating higher order structures is of paramount importance to utilize molecular organization in practical devices.

## Conflicts of interest

There are no conflicts to declare.

## Supplementary Material

Supplementary information
