# Supplementary material for: Impact of helical organization on the photovoltaic properties of oligothiophene supramolecular polymers
Source: Chem Sci. 2018 Mar 12;9(15):3638–43. doi: 10.1039/c7sc05093c (PMC5935057; doi:10.1039/c7sc05093c)
Supplement: Supplementary file 1 [file SC-009-C7SC05093C-s001.pdf]

Electronic Supplementary Material (ESI) for Chemical Science.
This journal is © The Royal Society of Chemistry 2018

Supporting Information

Impact of Helical Organization on Photovoltaic Properties of Oligothiophene Supramolecular Polymers

Hayato Ouchi,[a] Takahiro Kizaki,[b] Masaki Yamato,[c,d] Xu Lin,[e] Nagahiro Hoshi,[e] Fabien Silly,[f] Takashi Kajitani,[g,h] Takanori Fukushima,[g] Ken-ichi Nakayama,*[b,d] and Shiki Yagai*[e,i]

[a] Division of Advanced Science and Engineering, Graduate School of Science and Engineering, Chiba University, 1-33 Yayoi-cho, Inage-ku, Chiba 263-8522, Japan.

[b] Department of Organic Device Engineering, Graduate School of Science and Engineering, Yamagata University, 4-3-16 Jonan, Yonezawa, Yamagata 992-8510, Japan.

[c] Department of Organic Materials Science, Graduate School of Organic Materials Science, Yamagata University, 4-3-16 Jonan, Yonezawa, Yamagata 992-8510, Japan.

[d] Department of Material and Life Science, Graduate School of Engineering, Osaka University, 2-1 Yamadaoka, Suita, Osaka 565-0871, Japan.

[e] Department of Applied Chemistry and Biotechnology, Graduate School of Engineering, Chiba University, 1-33 Yayoi-cho, Inage-ku, Chiba 263-8522, Japan.

[f] TITANS, SPEC, CEA, CNRS, Université Paris-Saclay, CEA Saclay, F-91191 Gif sur Yvette, France.

[g] Laboratory for Chemistry and Life Science, Institute of Innovative Research, Tokyo Institute of Technology, 4259 Nagatsuta, Midori-ku, Yokohama 226-8503, Japan.

[h] RIKEN SPring-8 Center, 1-1-1 Kouto, Sayo, Hyogo 679-5148, Japan.

[i] Institute for Global Prominent Research (IGPR), Chiba University, 1-33 Yayoi-cho, Inage-ku, Chiba 263-8522, Japan.

Corresponding authors:

Shiki Yagai; E-mail: yagai@faculty.chiba-u.jp

Ken-ichi Nakayama; Email: nakayama@mls.eng.osaka-u.ac.jp

Table of Contents

1. General S2, S3
2. Synthesis and Analytical Data S4–S10
3. Supporting Figures S11–S14
4. Supporting Tables S15
5. References S16

1. General

Materials and Methods

Column chromatography was performed using 63–210 μm silica gel. Preparative gel permeation chromatography (GPC) was performed on a recycling preparative HPLC (LC-9225NEXT, Japan Analytical Industry) equipped with GPC columns JAIGEL-1H + 2H). All other commercially available reagents and solvents were of reagent grade and used without further purification. The solvents for the preparation of the assemblies were all spectral grade and used without further purification. ^{1}H and ^{13}C NMR spectra were recorded on Bruker DPS300 and JEOL JNM-ECA500 NMR spectrometers and chemical shifts are reported in ppm (δ) with the signal of TMS as internal standard. APCI- and ESI-MS spectra were measured on an Exactive (Thermo Fisher). Elemental analysis was performed in Chemical Analysis Center in Chiba University. TEM observation was performed on JEM-2100F (JEOL) at acceleration voltage at 120 kV. The sample was prepared by spin-coating assembly solution onto carbon-coated formvar copper grid (200 mesh) and dried under vacuum for 24 h. AFM images were acquired under ambient conditions using Multimode 8 Nanoscope V (Bruker AXS) in Peak Force Tapping (Scanasyst) mode. Silicon cantilevers (SCANASYST-AIR) with a spring constant of 0.4 N/m and a frequency of 70 kHz (Bruker AXS) were used. Synchrotron radiation X-ray diffraction measurements were performed at BL45XU in SPring-8 with the approval of the RIKEN SPring-8 Center (Proposal Nos. 20150068 and 20160027). Molecular mechanics calculations were performed on MacroModel version 10.4 using AMBER* force field. UV-vis spectra were recorded on a JASCO V660 spectrophotometer. Differential scanning calorimetry (DSC) was performed on SII DSC6220.

Scanning tunneling microscopy (STM)

1-Phenyloctane (98%, Aldrich) solutions of **3** and **4** were prepared at different concentrations. A droplet of these solutions was then deposited on a graphite substrate. STM imaging of the samples was performed at the liquid–solid interface using a Pico-SPM (Molecular Imaging, Agilent Technology) scanning tunneling microscope. Cut Pt/Ir tips were used to obtain constant current images at room temperature with a bias voltage applied to the sample. STM images were processed and analyzed using the application FabViewer.[1]

Fabrication of organic solar cells

Bulk heterojunction solar cell devices were fabricated on indium-tin oxide (ITO) coated glass. The ITO glass substrates were subsequently cleaned with acetone and 2-propanol in ultrasonic bath. The resultant ITO substrates were then exposed to UV-ozone for 20 min and coated with PEDOT:PSS [poly(3,4-ethylene dioxythiophene):poly(styrene sulfonate)](AI 4083, thickness: ca. 30 nm). The

substrates were heated for 20 min at 120 °C to remove residual water. In a N_2 glove box, 0.5 mL of chloroform solutions containing $PC_{71}BM$ (2.5 mg, Luminescence Technology Corp., Taiwan Province) and oligothiophene derivatives (2.5 mg) were spin-coated (1000 rpm for 30 s) onto the substrate. The thicknesses of the resulting BHJ films were determined to be 90–120 nm for **3**:$PC_{71}BM$ and 160–280 nm for **4**:$PC_{71}BM$, respectively, by DEKTAK surface profiler (Bruker AXS). Solvent vapor annealing (SVA) was conducted according to the method reported in the following reference: K. Sun, Z. Xiao, S. Lu, W. Zajaczkowski, W. Pisula, E. Hanssen, J. M. White, R. M. Williamson, J. Subbiah, J. Ouyang, et al., *Nat. Commun.* **2015**, *6*, 6013. The substrates were then moved to high-vacuum chamber, and the top electrode was evaporated through a shadow mask (Ca:10 nm, Al:90 nm) to give solar cells with an active area of 0.04 cm^2. Finally, the devices were encapsulated by a glass lid in the nitrogen glove box system. The *J*–*V* characteristics of the solar cells were evaluated by using a Keithley 2400 source-measure unit. The AM 1.5 G light was provided by a filtered Xe lamp. The intensity of 100 mW cm^{-2} of the AM 1.5 G light was determined by using a calibrated inorganic solar cell from the National Institute of Advanced Industrial Science and Technology (Japan). No spectral mismatch factor was in the calculation of the efficiency.

Hole mobility measurements

Hole mobilities of **3**:$PC_{71}BM$ and **4**:$PC_{71}BM$ (prepared from 10 mg mL^{-1} $CHCl_3$ solutions) without and with SVA using CS_2 were estimated by the charge-only space-charge limited current (SCLC) method. The SCLC is described by $J = 9\varepsilon_0\varepsilon_r\mu V^2/8L^3$, where J is the current density, L is the film thickness of the active layer, μ is the hole mobility, ε_r is the relative dielectric constant of the transport medium, ε_0 is the permittivity of free space (8.85×10^{-12} F m^{-1}), V is the applied voltage to the device. SCLC measurements were carried out with a device structure of ITO/MoO_3/active layer/MoO_3/Al by taking the current density in the range 0–10 V and fitting the results to a space-charge limited form.

2. Synthesis and Analytical Data

Compound **3** and **4** were synthesized according to Scheme S1. The syntheses of reference compounds **1** and **2** were reported previously.[2,3]

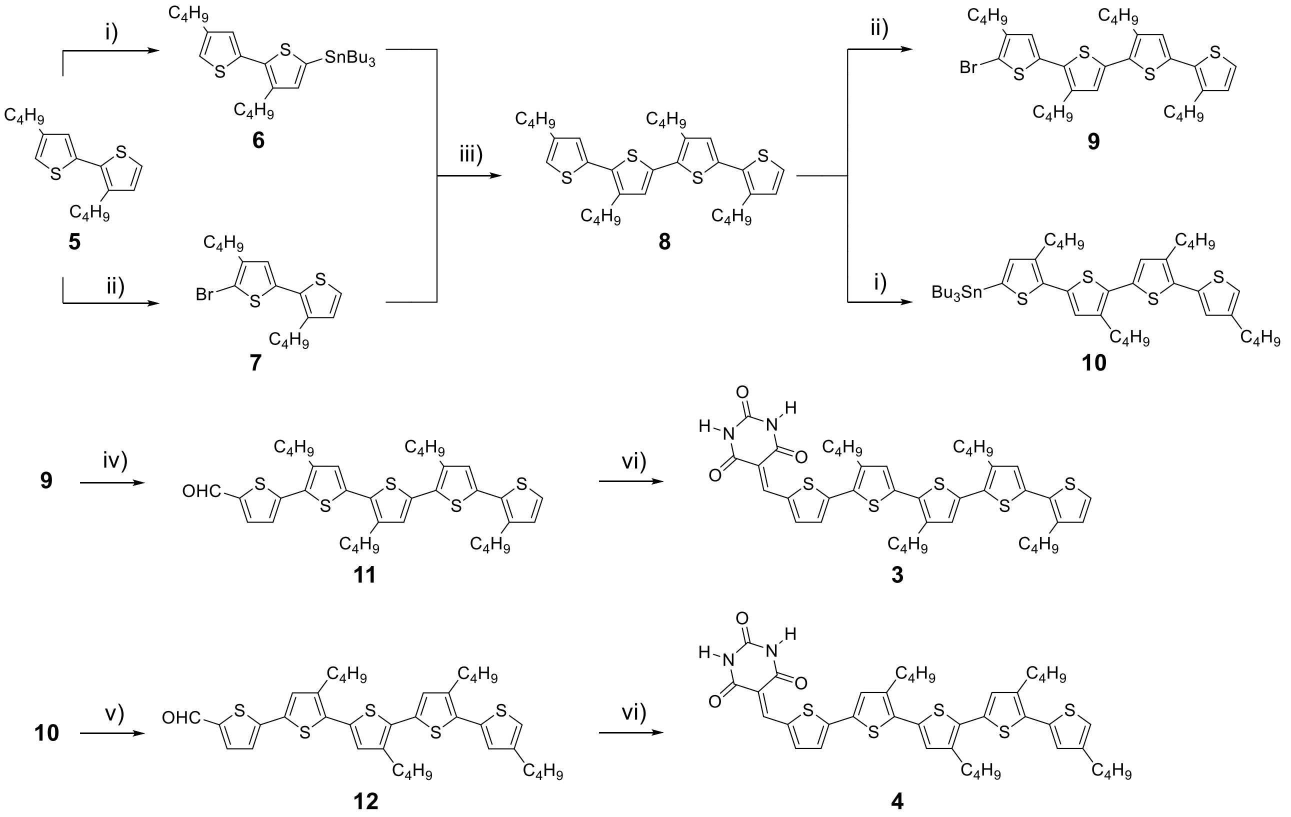

Scheme S1. Synthesis of **3** and **4**. i) LDA, $SnBu_3Cl$, THF, –78 °C→r.t.; ii) NBS, $CHCl_3$/acetic acid, 0 °C→r.t.; iii) $[Pd(PPh_3)_4]$, DMF, microwave, 150 °C; iv) 5-formylthiophene-2-boronic acid, K_2CO_3, $Pd(dppf)Cl_2$, toluene/MeOH, 80 °C; v) 5-bromothiophene-2-carbaldehyde, $[Pd(PPh_3)_4]$, DMF, microwave, 150 °C; vi) barbituric acid, EtOH, reflux.

Tributyl(3,4'-dibutyl-[2,2'-bithiophen]-5-yl)stannane (6): To a dry THF (50 mL) solution of **5** (1.25 g, 4.52 mmol) cooled to –78 °C, a hexane/THF solution of lithium diisopropylamide (1.13 M, 4 mL, 4.52 mmol) was dropped over 10 min and the mixture was stirred at –78 °C for 1 h under N_2 atmosphere. After 1 h, $SnBu_3Cl$ (1.50 g, 4.61 mmol) was added and the mixture was stirred for 30 min. The reaction mixture was warmed to r.t., and further stirred overnight. The reaction mixture was poured into water, and evaporated to remove THF. The resulting mixture was extracted by $CHCl_3$, and washed twice with water. The organic layer was dried with Na_2SO_4, filtered and evaporated to dryness to give compound **6** as light yellow liquid (2.49 g). This compound was used for the following reaction without purification.

5'-Bromo-3,4'-dibutyl-2,2'-bithiophene (7): To an ice-cooled 2:1 $CHCl_3$/acetic acid mixture (30 mL) containing **5** (750 mg, 2.69 mmol), *N*-bromosuccinimide (478 mg, 2.69 mmol) was added in three portions at interval of 10 min. The reaction mixture was warmed to r.t. and stirred for 3 h. The resulting mixture was diluted with $CHCl_3$, and washed twice with sat. aq. $NaHCO_3$. The organic layer was dried with Na_2SO_4, filtered and evaporated to dryness to give compound **7** as light yellow liquid (934 mg, 97%). ^{1}H NMR (300 MHz, $CDCl_3$, 293 K): δ = 7.16 (d, J = 5.2 Hz, 1H), 6.91 (d, J = 5.2 Hz, 1H), 6.79 (s, 1H), 2.71 (t, J = 7.7 Hz, 2H), 2.61 (t, J = 7.7 Hz, 2H), 1.65–1.54 (m, 4H), 1.44–1.32 (m, 4H), 0.97–0.90 (m, 6H); HRMS (APCI): *m/z* calcd. for $C_{16}H_{22}BrS_2$ 357.0341 $[M+H]^+$, found 357.0335.

3,4',4'',4'''-Tetrabutyl-2,2':5',2'':5'',2'''-quaterthiophene (8): A 5-mL of microwave reactor vessel was charged with **6** (2.04 g, 3.59 mmol), **7** (865 mg, 2.42 mmol), $Pd(PPh_3)_4$ (45.6 mg, 0.0395 mmol) and DMF (3 mL), and the vessel was placed into a microwave reactor. The reaction temperature and period were set to 150 °C and 30 min, respectively. After completion of the reaction, the mixture was diluted with ethyl acetate and washed twice with water and once with brine. The organic layer was dried with Na_2SO_4, filtered and evaporated to dryness. The resulting liquid was purified by column chromatography (10 w/w% K_2CO_3-silica gel, eluent: hexane) to give pure compound **8** as yellow liquid (1.23 g, 92%). ^{1}H NMR (300 MHz, $CDCl_3$, 293 K): δ = 7.16 (d, J = 5.2 Hz, 1H), 6.97 (s, 1H), 6.95 (s, 1H), 6.94 (s, 1H), 6.93 (d, J = 5.2 Hz, 1H), 6.90 (s, 1H), 2.81–2.73 (m, 6H), 2.62 (t, J = 7.7 Hz, 2H), 1.72–1.58 (m, 8H), 1.49–1.33 (m, 8H), 0.98–0.92 (m, 12H); HRMS (ESI): *m/z* calcd. for $C_{32}H_{42}S_4$ 555.2242 $[M+H]^+$, found 555.2235.

5'''-Bromo-3,4',4'',4'''-tetrabutyl-2,2':5',2'':5'',2'''-quaterthiophene (9): To an ice-cooled 2:1 $CHCl_3$/acetic acid mixture (30 mL) containing **8** (885 mg, 1.59 mmol), *N*-bromosuccinimide (283 mg, 1.59 mmol) was added in three portions with interval of 10 min. The mixture was warmed to r.t. and stirred for 3 h. The reaction mixture was diluted with $CHCl_3$, washed twice with sat. aq. $NaHCO_3$. The organic layer was dried with Na_2SO_4, filtered and evaporated to dryness. The residual liquid was purified by column chromatography (silica gel, eluent: hexane) to give pure compound **9** as yellow liquid (727 mg, 72%). ^{1}H NMR (300 MHz, $CDCl_3$, 293 K): δ = 7.15 (d, J = 5.2 Hz, 1H), 6.94 (s, 1H), 6.93 (s, 1H), 6.92 (d, J = 5.2 Hz, 1H), 6.82 (s, 1H), 2.81–2.69 (m, 6H), 2.57 (t, J = 7.7 Hz, 2H), 1.71–1.55 (m, 8H), 1.49–1.35 (m, 8H), 0.98–0.92 (m, 12H); HRMS (APCI): *m/z* calcd. for $C_{32}H_{42}BrS_4$ 633.1347 $[M+H]^+$, found 633.1339.

Tributyl(3,4',4'',4'''-tetrabutyl-[2,2':5',2'':5'',2'''-quaterthiophen]-5-yl)stannane (10): To a dry THF (30 mL) solution of **8** (630 mg, 1.14 mmol) cooled to –78 °C, a hexane/THF solution of lithium diisopropylamide (1.13 M, 1.3 mL, 1.47 mmol) was dropped over 10 min and the mixture

was stirred at –78 °C for 1 h under N_2 atmosphere. After 1 h, $SnBu_3Cl$ (405 mg, 1.24 mmol) was added and the mixture was stirred for 30 min. The reaction mixture was warmed to r.t., and further stirred overnight. The reaction mixture was poured into water, and evaporated to remove THF. The resulting mixture was extracted by $CHCl_3$, and washed twice with water. The organic layer was dried with Na_2SO_4, filtered and evaporated to dryness to give compound **10** as yellow liquid (2.49 g). This compound was used for the following reaction without purification.

3',3'',3''',3''''-Tetrabutyl-[2,2':5',2'':5'',2''':5''',2''''-quinquethiophene]-5-carbaldehyde (11): To a mixture of **9** (892 mg, 1.40 mmol) and 5-formylthiophene-2-boronic acid (438 mg, 2.80 mmol) in 1:1 toluene/methanol mixture (20 mL), Pd(dppf)Cl_2 (52.0 mg, 0.0710 mmol), K_2CO_3 (976 mg, 7.07 mmol) were added and the resulting mixture was stirred at 80 °C for 11 h under N_2 atmosphere. The reaction mixture was cooled to r.t., diluted with ethyl acetate, and then washed twice with water. The organic layer was dried with Na_2SO_4, filtered and evaporated to dryness. The residual liquid was purified by column chromatography (silica gel, eluent: $CHCl_3$/hexane = 1:1 (v/v)) to give pure compound **11** as orange liquid (478 mg, 51%). ^{1}H NMR (500 MHz, $CDCl_3$, 293 K): δ = 9.88 (s, 1H), 7.70 (d, J = 3.9 Hz, 1H), 7.24 (d, J = 3.9 Hz, 1H), 7.16 (d, J = 5.1 Hz, 1H), 7.01 (s, 1H), 6.97 (s, 1H), 6.95 (s, 1H), 6.93 (d, J = 5.2 Hz, 1H), 2.85–2.78 (m, 8H), 1.73–1.62 (m, 8H), 1.50–1.38 (m, 8H), 0.99–0.94 (m, 12H); ^{13}C NMR (125 MHz, $CDCl_3$, 293 K): δ = 182.68, 146.32, 142.62, 142.15, 140.70, 140.08, 139.74, 137.04, 136.31, 134.62, 134.46, 130.51, 130.15, 129.71, 129.33, 129.00, 128.89, 128.76, 125.91, 123.79, 33.95, 32.78, 32.75, 32.53, 29.65, 29.38, 29.30, 29.15, 22.82, 14.12; HRMS (ESI): m/z calcd for $C_{37}H_{44}OS_5$ 665.2068 $[M+H]^+$, found 665.2063.

4',4'',4''',4''''-Tetrabutyl-[2,2':5',2'':5'',2''':5''',2''''-quinquethiophene]-5-carbaldehyde (12): A 5-mL of microwave reactor vessel was charged with **10** (942 mg, 1.12 mmol), 5-bromothiophene-2-carbaldehyde (156 mg, 0.817 mmol), $Pd(PPh_3)_4$ (27.0 mg, 0.0234 mmol) and DMF (4 mL), and the vessel was placed into a microwave reactor. The reaction temperature and period was set to 150 °C and 15 min, respectively. After completion of the reaction, the mixture was diluted with ethyl acetate and washed twice with water and once with brine. The organic layer was dried with Na_2SO_4, filtered and evaporated to dryness. The resulting liquid was purified by column chromatography (silica gel, eluent: $CHCl_3$/hexane = 1:1 (v/v)) to give pure compound **12** as orange liquid (219 mg, 40%). ^{1}H NMR (500 MHz, $CDCl_3$, 293 K): δ = 9.84 (s, 1H), 7.65 (d, J = 3.9 Hz, 1H), 7.20 (d, J = 3.9 Hz, 1H), 7.18 (s, 1H), 7.00 (s, 1H), 6.98 (s, 1H), 6.97 (s, 1H), 6.90 (s, 1H), 2.80–2.75 (m, 6H), 2.64–2.61 (t, J = 7.7 Hz, 2H), 1.70–1.61 (m, 8H), 1.48–1.36 (m, 8H), 0.99–0.94 (m, 12H); ^{13}C NMR (125 MHz, $CDCl_3$, 293 K): δ = 182.50, 147.11, 143.74, 141.51, 140.70, 139.91, 139.69, 137.56, 135.45, 133.24, 133.21, 132.94, 132.89, 131.56, 131.43, 129.31, 129.26, 128.79, 127.34, 124.01, 120.22, 33.95, 32.88, 32.80, 32.70, 30.31, 29.24, 29.12, 22.79, 22.53, 14.10; HRMS

(ESI): *m/z* calcd for $C_{37}H_{44}OS_5$ 665.2068 $[M+H]^+$, found 665.2068.

Compound 3: A mixture of **11** (154 mg, 0.231 mmol) and barbituric acid (158 mg, 1.23 mmol) in EtOH (3 mL) was refluxed for 4 h. The reaction mixture was cooled to r.t., and the resulting precipitates were collected by filtration and washed with hot water to give nearly pure compound **3** as a black-purple solid (38.0 mg, 21%). This compound was further purified by preparative gel permeation chromatography (GPC) using $CHCl_3$ as eluent. ^{1}H NMR (500 MHz, $CDCl_3$, 293 K): δ = 8.61 (s, 1H), 8.55 (br-s, 1H), 8.29 (br-s, 1H), 7.88 (d, *J* = 4.2 Hz, 1H), 7.37 (d, *J* = 4.2 Hz, 1H), 7.17 (d, *J* = 5.2 Hz, 1H), 7.04 (s, 1H), 6.97 (s, 1H), 6.94 (s, 1H), 6.93 (d, *J* = 5.2 Hz, 1H), 2.94 (t, *J* = 7.7 Hz, 2H), 2.82–2.77 (m, 6H), 1.77–1.63 (m, 8H), 1.52–1.38 (m, 8H), 1.00–0.93 (m, 12H); HRMS (APCI): *m/z* calcd for $C_{41}H_{46}O_3N_2S_5$ 775.2185 $[M+H]^+$, found 775.2167; Elemental analysis: calcd for $C_{41}H_{46}O_3N_2S_5$ (%): C, 63.53; H, 5.98; N, 3.61. Found: 63.36; H, 5.85; N, 3.61. ^{13}C NMR spectrum of this compound could not be measured due to its low solubility.

Compound 4: A mixture of **12** (106 mg, 0.159 mmol) and barbituric acid (106 mg, 0.828 mmol) in EtOH (30 mL) was refluxed for 3 h. The reaction mixture was cooled to r.t., and the resulting precipitates were collected by filtration and washed with hot water to give nearly pure compound **4** as a black-purple solid (94.3 mg, 77%). ^{1}H NMR (500 MHz, $(CD_3)_2SO$, 293 K): δ = 11.3 (s, 2H), 8.47 (s, 1H), 8.14 (d, *J* = 4.2 Hz, 1H), 7.61 (d, *J* = 4.2 Hz, 1H), 7.60 (s, 1H), 7.22 (s, 1H), 7.22 (s, 1H), 7.13 (s, 1H), 7.08 (s, 1H), 2.94 (t, *J* = 7.7 Hz, 2H), 2.81–2.72 (m, 6H), 2.59 (t, *J* = 7.6 Hz, 2H), 1.68–1.55 (m, 8H), 1.44–1.29 (m, 8H), 0.95–0.69 (m, 12H); HRMS (ESI): *m/z* calcd for $C_{41}H_{46}O_3N_2S_5$ 775.2185 $[M+H]^+$, found 775.2183; Elemental analysis: calcd for $C_{41}H_{46}O_3N_2S_5$ (%): C, 63.53; H, 5.98; N, 3.61. Found: 62.85; H, 5.81; N, 3.56. ^{13}C NMR spectrum of this compound could not be measured due to its very low solubility.

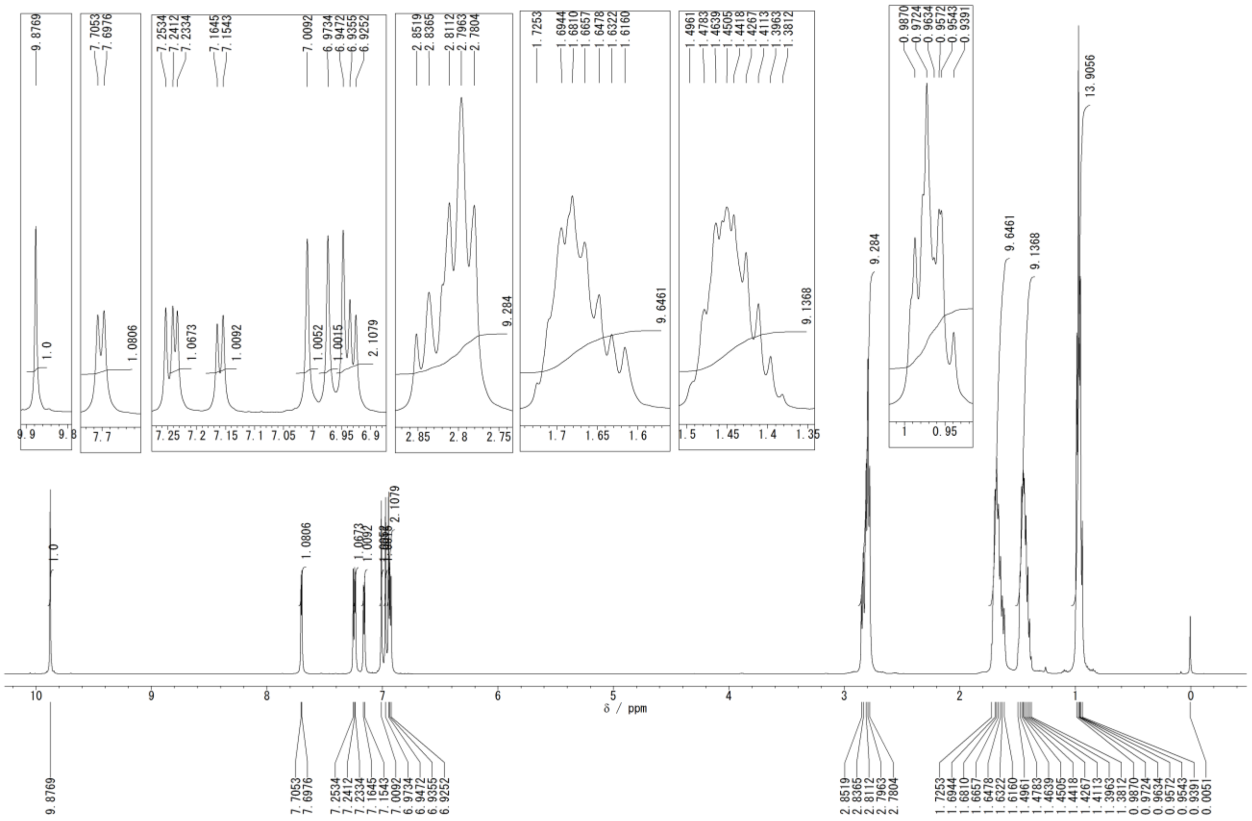

Chart S1. ^{1}H NMR spectrum of **11** in $CDCl_3$ at 293 K.

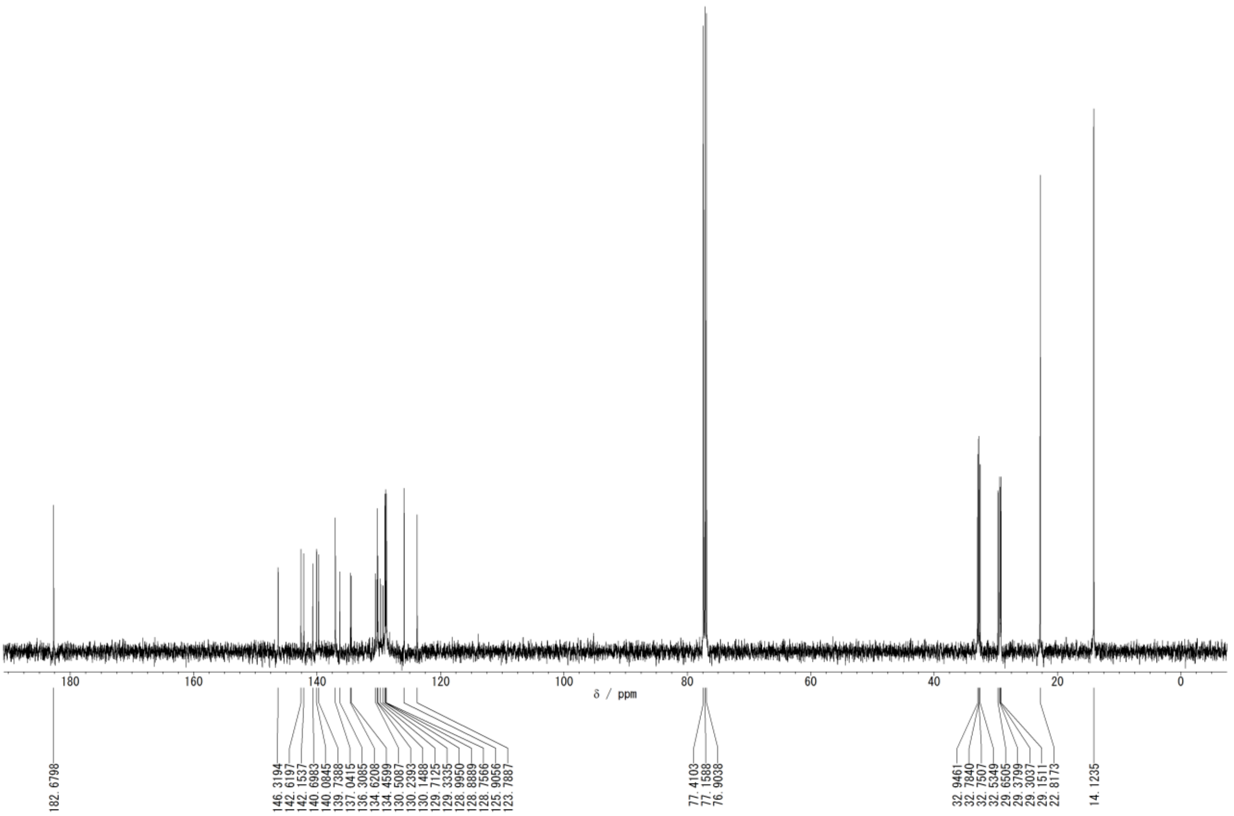

Chart S2 ^{13}C NMR spectrum of **11** in $CDCl_3$ at 293 K.

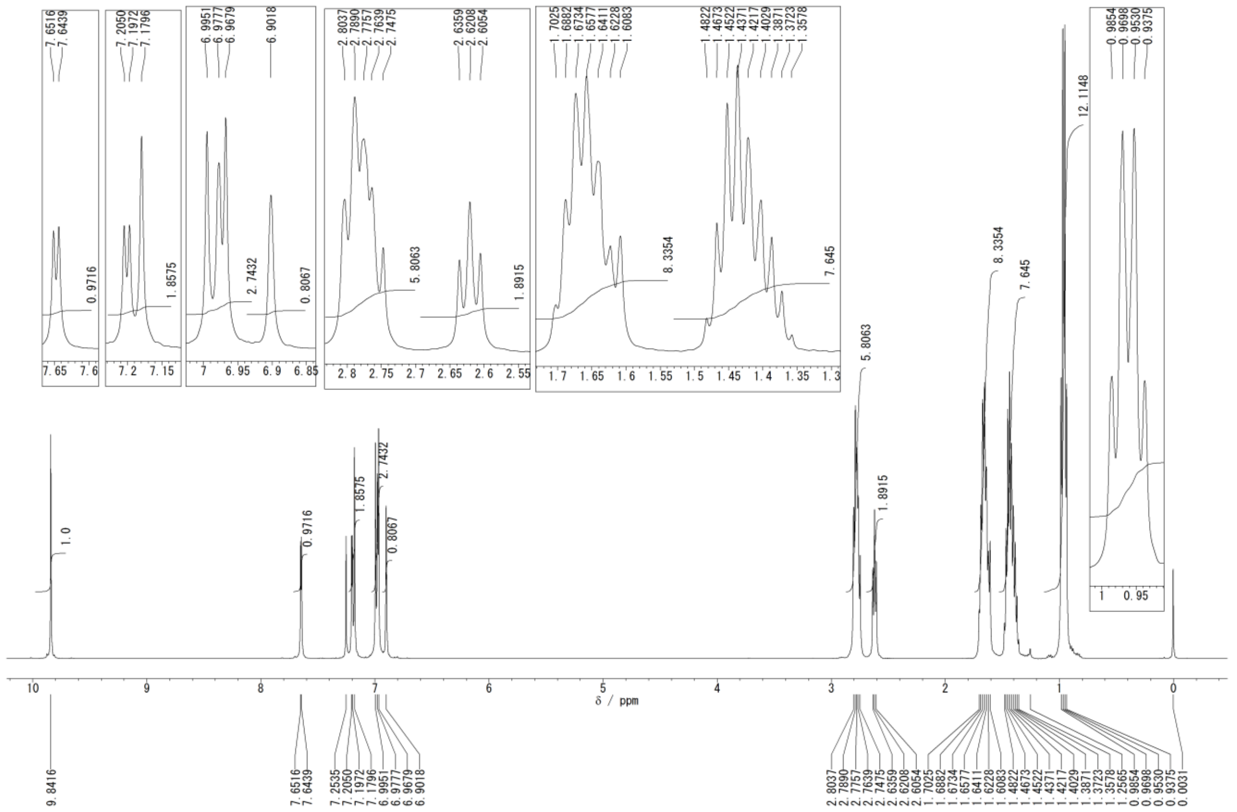

Chart S3 ^{1}H NMR spectrum of **12** in $CDCl_3$ at 293 K.

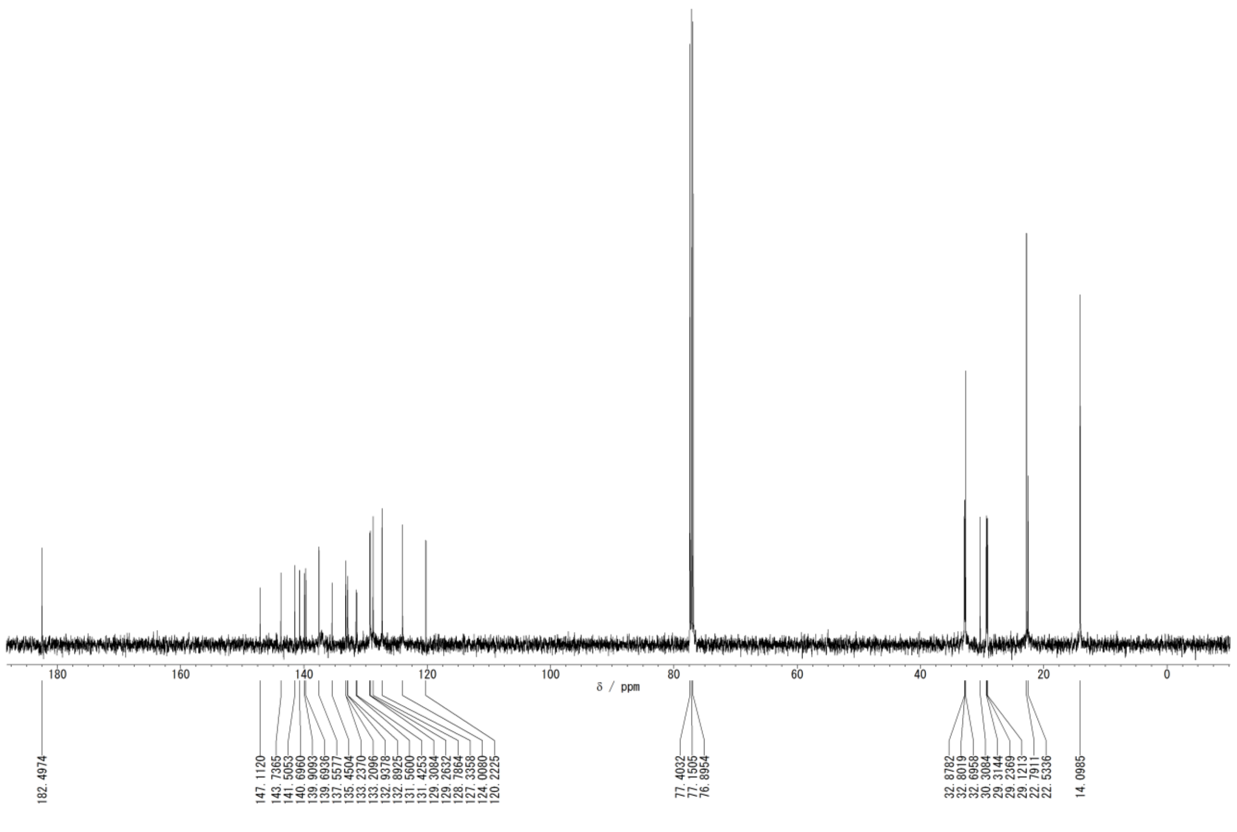

Chart. S4 ^{13}C NMR spectrum of **12** in $CDCl_3$ at 293 K.

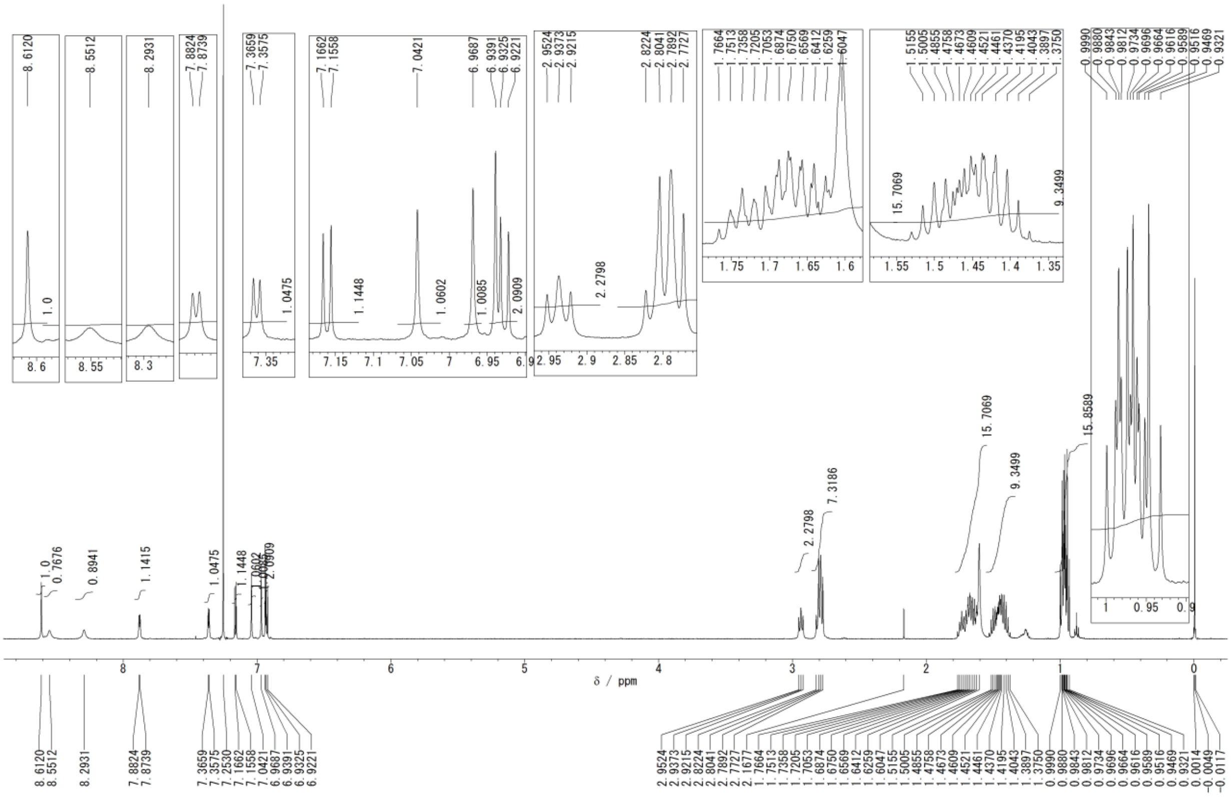

Chart S5. ^{1}H NMR spectrum of **3** in $CDCl_3$ at 293 K.

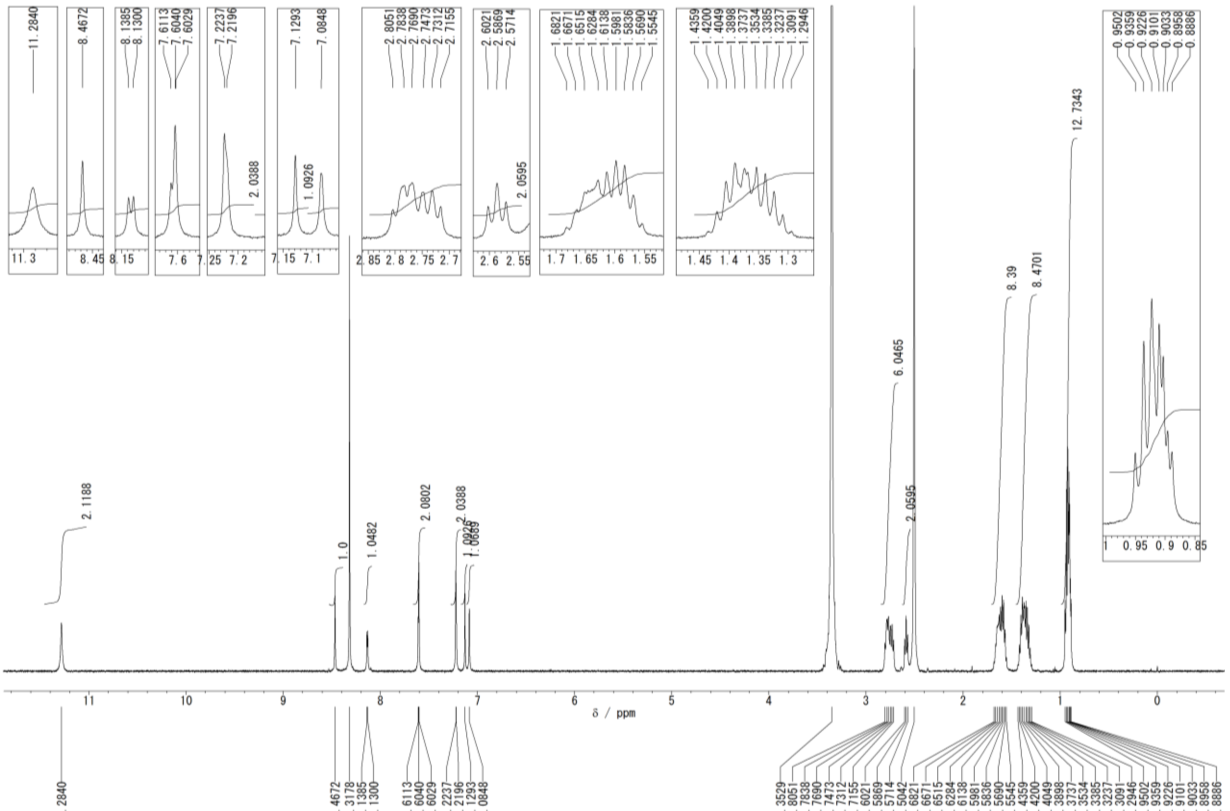

Chart S6 ^{1}H NMR spectrum of **4** in $(CD_3)_2SO$ at 293 K.

3. Supporting Figures

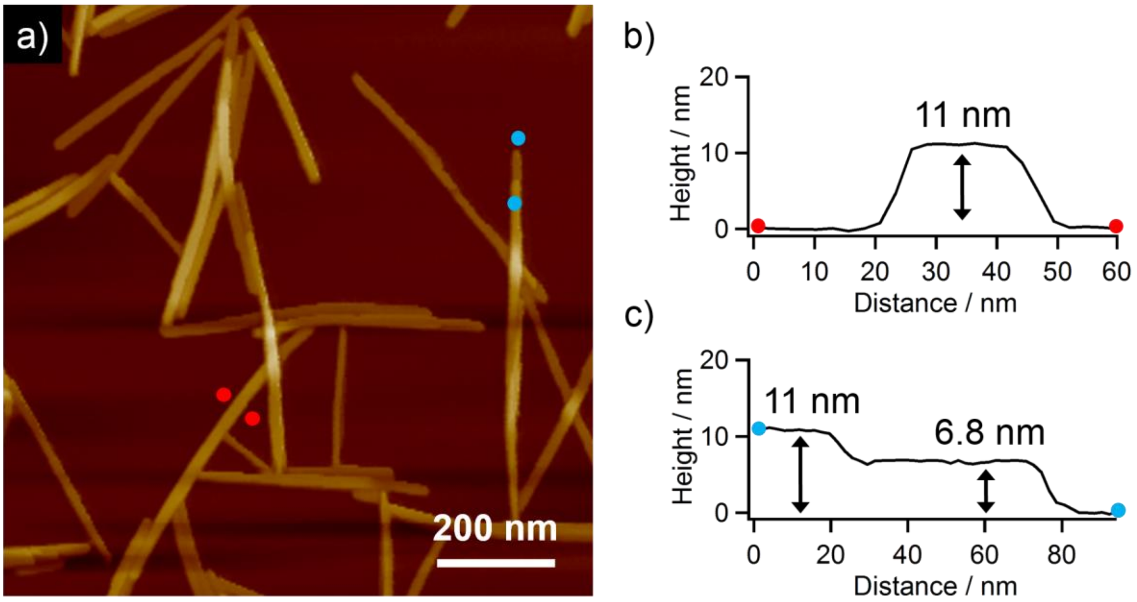

Fig. S1. (a) AFM image of thin film prepared by drop-casting toluene solution of **4** ($c = 1 \times 10^{-4}$ M) onto HOPG. (b,c) Cross-sectional analysis between (b) red and (c) blue dots (for height) in the image (a).

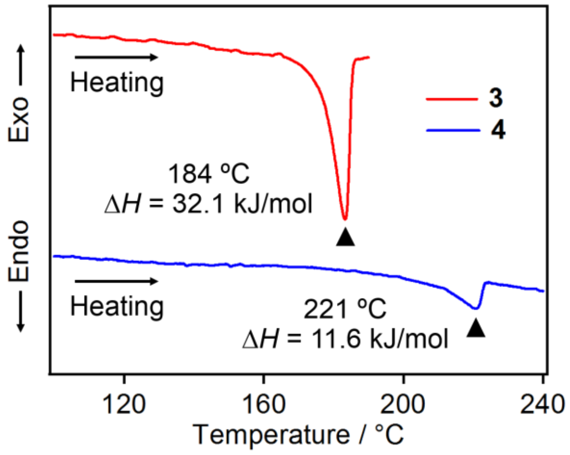

Fig. S2. DSC profiles (second heating) of **3** (red) and **4** (blue) measured at a scan rate of 3 ºC/min. All samples were prepared by drop-casting $CHCl_3$ solutions onto a glass plate to form thin films. After drying at room temperature for 24 h to remove the residual solvent, the films were collected by scratching and transferred to an aluminum sample pan for DSC measurements.

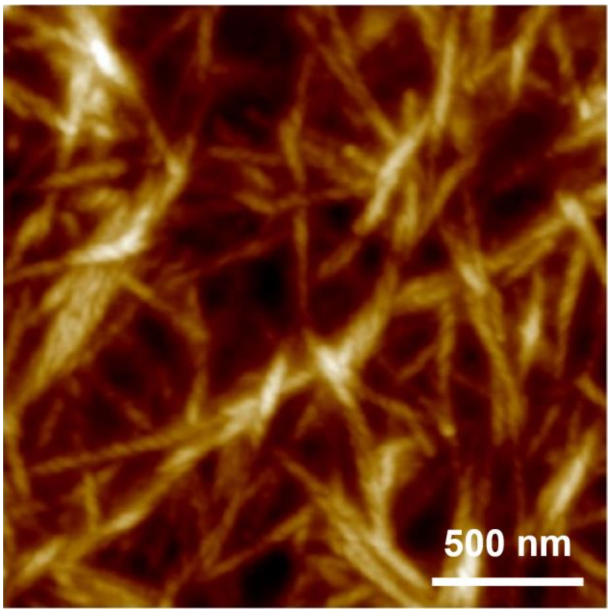

Fig. S3. AFM image of helical fibers formed in **3**:$PC_{61}BM$ film. The film was prepared by spin-coating a chloroform solution of the 1:1 mixture (c_{total} = 10 mg mL^{-1}) and thermal annealing the resulting film at 110 ºC.

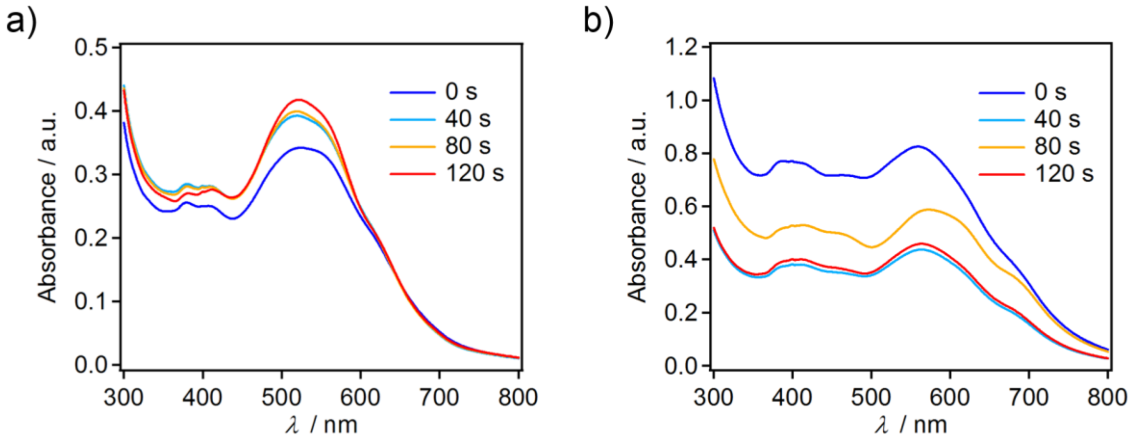

Fig. S4. (a,b) Effect of SVA (0, 40, 80 ad 120 sec) using CS_2 on UV-vis spectra of BHJ solar cells based on (a) **3**:$PC_{71}BM$ and (b) **4**:$PC_{71}BM$. Film thickness: 90–120 nm for **3**:$PC_{71}BM$; 160–280 nm for **4**:$PC_{71}BM$.

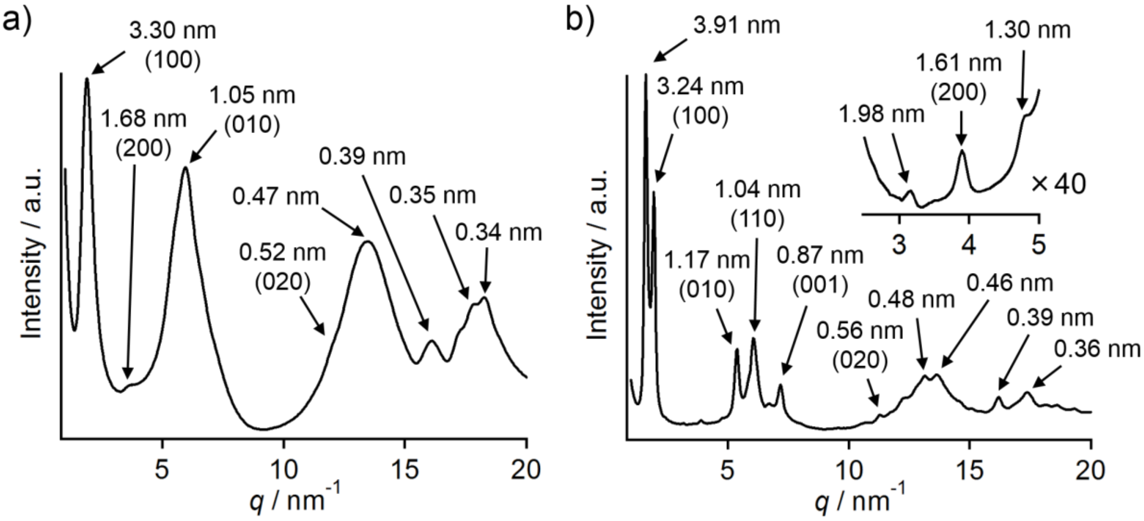

Fig. S5. (a,b) PXRD patterns of bulk sample of (a) **3**:$PC_{71}BM$ and (b) **4**:$PC_{71}BM$ in a glass capillary (diameter: 1.0 mm). Values in parenthesis denote Miller indices. The four peaks in small-angle region in (a) can be thus assigned to the diffractions from the (100), (200), (010), and (020) planes of a 2D rectangular lattice (space group: *P*2*m*, lattice parameters: *a* = 3.2 nm, *b* = 1.2 nm), respectively. Likewise, six peaks in small-angle region in (b) can be thus assigned to the diffractions from the (100), (200), (010), (110), (001), and (020) planes of a 2D rectangular lattice (space group: *P*2*m*, lattice parameters: *a* = 3.2 nm, *b* = 1.2 nm), respectively.

Note: The sharp diffraction at *d* = 3.91 nm, which is accompanied by the higher order diffractions at *d* = 1.98 and 1.30 nm, cannot be assigned at the moment. Because the intensities of these diffraction peaks differ by samples, they do not arise from the structural rearrangement of **4**. They may rather arise from certain ordering of $PC_{71}BM$ upon co-aggregation with highly crystalline **4**.

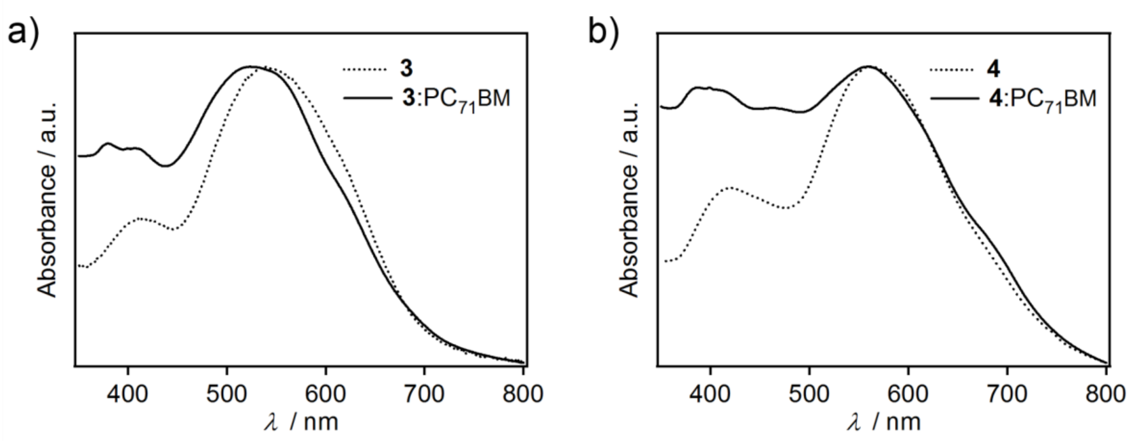

Fig. S6. (a,b) Normalized UV-vis spectra of thin films of (a) **3** (dashed curve, c = 5 mg mL^{-1}) and **3**:PC$_{71}$BM (solid curve, c_{total} = 10 mg mL^{-1}), and (b) **4** (dashed curve, c = 5 mg mL^{-1}) and **4**:PC$_{71}$BM (solid curve, c_{total} = 10 mg mL^{-1}).

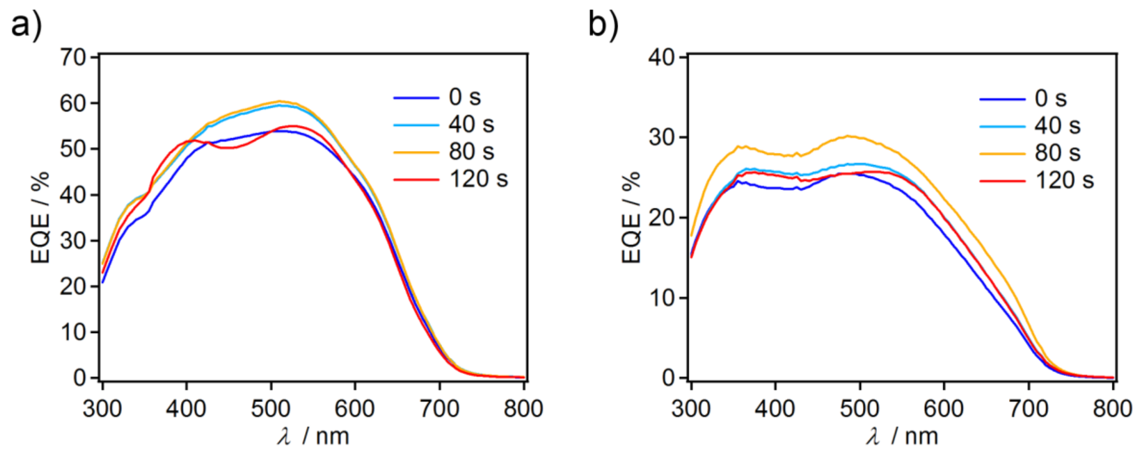

Fig. S7. (a,b) Effect of SVA (0, 40, 80 and 120 sec) using CS_2 on EQE spectra of BHJ solar cells based on (a) **3**:PC$_{71}$BM and (b) **4**:PC$_{71}$BM.

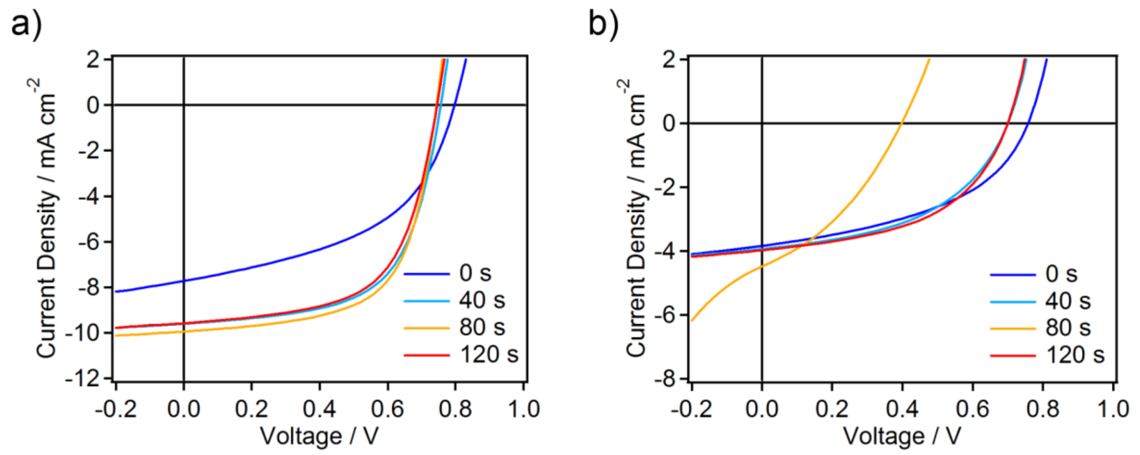

Fig. S8. (a,b) Effect of SVA (0, 40, 80 and 120 sec) using CS_2 on J–V characteristics of (a) **3**:PC$_{71}$BM and (b) **4**:PC$_{71}$BM BHJ solar cells.

4. Supporting Tables

Table S1. Effect of thermal annealing on photovoltaic properties of **3**:$PC_{71}BM$ and **4**:$PC_{71}BM$ BHJ solar cells.

BHJ films	Annealing temp [s]	J_{sc} [mA cm^{-2}]	V_{oc} [V]	FF [%]	PCE [%]
**3**:$PC_{71}BM$	As-cast	6.63 ± 0.11	0.86 ± 0.02	36.9 ± 0.7	2.10 ± 0.09
	80	5.44 ± 0.09	0.81 ± 0.03	34.0 ± 1.4	1.49 ± 0.14
	110	6.13 ± 0.19	0.71 ± 0.01	36.3 ± 0.4	1.57 ± 0.04
	130	6.13 ± 0.02	0.69 ± 0.01	43.5 ± 0.7	1.82 ± 0.05
**4**:$PC_{71}BM$	As-cast	3.53 ± 0.23	0.75 ± 0.01	45.2 ± 0.2	1.19 ± 0.10
	80	3.56 ± 0.14	0.76 ± 0.01	49.3 ± 0.6	1.33 ± 0.06
	110	0.81 ± 0.36	0.74 ± 0.01	41.9 ± 1.1	0.26 ± 0.12
	130	1.12 ± 0.16	0.75 ± 0.01	43.4 ± 2.7	0.36 ± 0.04

Table S2. Hole mobilities of **3**:$PC_{71}BM$ and **4**:$PC_{71}BM$ without and with SVA (CS_2) evaluated by the charge-only space-charge limited current (SCLC) method.

BHJ films	SVA time [s]	Thickness [nm]	Hole mobility [cm^2 V^{-1} s^{-1}]
**3**:$PC_{71}BM$	0	115	6.6×10^{-6}
	80	107	1.2×10^{-5}
**4**:$PC_{71}BM$	0	172	1.2×10^{-3}
	80	198	4.3×10^{-3}

5. References

1 F. Silly, *J. Microsc-Oxford*, **2009**, *236,* 211–218.

2 S. Yagai, M. Suzuki, X. Lin, M. Gushiken, T. Noguchi, T. Karatsu, A. Kitamura, A. Saeki, S. Seki, Y. Kikkawa, Y. Tani, and K. Nakayama, *Chem. Eur. J.* **2014**, *20*, 16128–16137.

3 X. Lin, M. Suzuki, M. Gushiken, M. Yamauchi, T. Karatsu, T. Kizaki, Y. Tani, K. Nakayama, M. Suzuki, H. Yamada, T. Kajitani, T. Fukushima, Y. Kikkawa, and S. Yagai, *Sci. Rep.* **2017**, *7*, 43098.
